# Legionellosis: Diagnosis and Control in the Genomic Era

**DOI:** 10.3201/eid2802.212055

**Published:** 2022-02

**Authors:** Paul H. Edelstein

**Affiliations:** University of Pennsylvania, Philadelphia, Pennsylvania, USA; University of Cambridge, Cambridge, UK

**Keywords:** legionellosis, bacteria, respiratory infections, *Legionella pneumophila*, whole-genome sequencing

Hundreds of books and textbook chapters, and thousands of journal review articles, have been published on Legionnaires’ disease and *Legionella* spp. bacteria over the past 45 years, making it important to decide whether this new and quite expensive compilation of reviews is worth acquiring (Figure). The field has become so specialized that even those who know one aspect of it may need a good review of other aspects to easily catch up on recent trends. The book contains chapters on the freshwater ecology of the bacterium; molecular and pathogenic aspects of virulence-associated bacterial secretion systems; very selected aspects of epidemiology; clinical aspects and treatment; laboratory diagnosis; and strain typing methods from serologic to whole-genome sequencing. Some chapters are more current than others. The most recent references for several chapters were published in 2016, and only 1 chapter cites references published in 2020. The book is lightly edited; some of the chapters contain overlapping material, but overall it has few typographical or spelling errors. Not all of the figures are properly labeled; for example, the figure legends in chapter 6 are reversed, and not all of the figure legends in chapter 3 fully explain the meanings of different colors and abbreviations.

**Figure:**
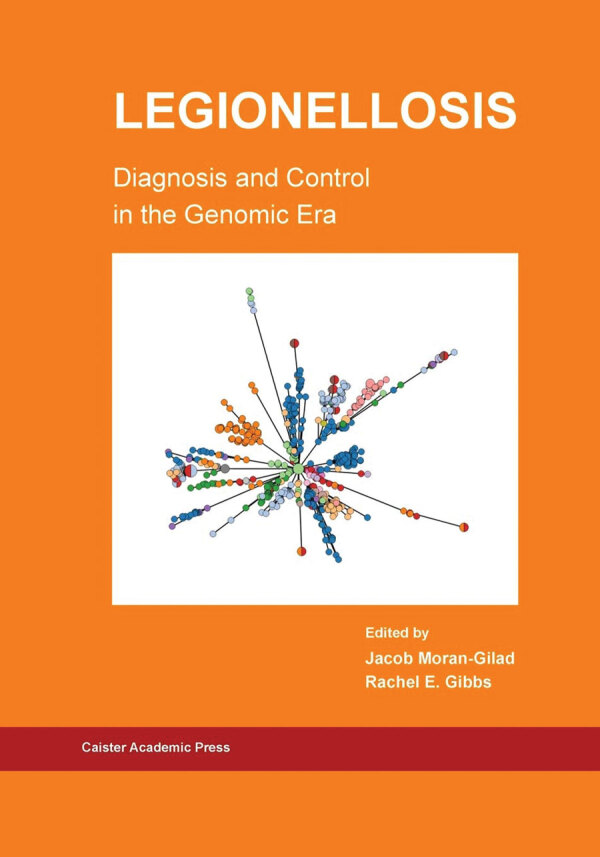
Legionellosis: Diagnosis and Control in the Genomic Era

I found that several of the chapters contained quite useful information that would be hard to find elsewhere, including a thorough review of *L. pneumophila* virulence secretory systems, as well as a review of the freshwater ecology of the bacterium, the clinical microbiology and clinical significance of *Legionella* spp. other than *L. pneumophila*, and regulatory and risk management strategies for control of the disease. Other readers, depending on their fields of interest and expertise, will find other chapters of particular interest. The chapter on non–whole-genome sequencing methods for strain typing for epidemiologic investigation is well done and could be of interest for those trying to dissect the older literature. Missing from the book, presumably by design, are a chapter reviewing in detail the ecology of the bacterium in the built environment, practical guidance on outbreak investigation, advanced techniques in epidemiologic source investigation, molecular and cellular pathogenesis other than secretion systems, and the molecular evolution of the bacterium, all of which can be found in other sources.

Is this book good value for money? Perhaps not for those who have a narrow interest in a specific field, because there are more up-to-date reviews on many of the topics in journal articles and some textbooks. For those who want to gain an overview of the topics covered in the book, some of which are more comprehensive than those found in textbooks or recent reviews, this may be a useful addition to their libraries.

